# Performance Characteristics of Silicone Rubber for Use in Acidic Environments

**DOI:** 10.3390/polym15173598

**Published:** 2023-08-30

**Authors:** Zhijin Zhang, Zhiqin Zhang, Song Yue, Xingliang Jiang, Jianlin Hu

**Affiliations:** Xuefeng Mountain Energy Equipment Safety National Observation and Research Station, Chongqing University, Chongqing 400044, China; zhangzqin@stu.cqu.edu.cn (Z.Z.); yuesong@stu.cqu.edu.cn (S.Y.); xljiang@cqu.edu.cn (X.J.); hujianlin@cqu.edu.cn (J.H.)

**Keywords:** silicone rubber, composite insulators, acidic environment, acid-resistant silicone rubber, performance characteristics

## Abstract

Silicone rubber insulators are widely used in power grids because of their excellent performance, but aging has been an inevitable problem of silicone rubber, especially in extreme conditions, such as acidic conditions. In order to clarify the performance changes in silicone rubber in an acidic environment, this paper uses the developed acid-resistant silicone rubber sheet and common silicone rubber samples as the research objects, and conducts an aging comparison test on them in a natural acidic environment. The electrical properties, physical properties, and chemical properties of the two types of silicone rubber specimens with different aging times are analyzed to obtain the performance characteristics of silicone rubber under a natural acidic environment. The research results show that the dry flash voltage and pollution flashover voltage of the acid-resistant silicone rubber after one year of aging are greater than those of the common type; the water repellency of both types of silicone rubber remains in good condition. The silicone rubber produced by our team according to the self-developed acid-resistant silicone rubber formula has indeed played a role in delaying aging in an acidic environment compared with the common-type silicone rubber.

## 1. Introduction

Silicone rubber composite insulators were first used as a solution to pollution flashover events due to their excellent antifouling properties, and then used in power grids on a large scale due to their excellent electrical and mechanical properties; nowadays, China’s composite insulators account for more than half of the new insulator market [1,2,3,4].

With the operation of silicone rubber under electricity, the characteristics of silicone rubber polymers determine the inevitable aging problems [5,6]. Regarding the detection of the degree of aging of silicone rubber, there has been a shift from destructive tests, such as leakage current and flashover voltage [7], before 2010 to nondestructive testing methods based on the principle of nuclear magnetic resonance around 2015. This is represented by the unilateral nuclear magnetic resonance (NMR) detection technique, a portable arc-shaped NMR sensor used by Xu Z et al. [8,9]. The latest research, around 2020, is mainly based on microscopic-image techniques and the photothermal radiation (PTR) method. For example, Qiao X et al. proposed a new method based on microscopic images to evaluate the aging performance of silicone rubber insulators, and also showed its future-use scenarios [10]; Jiang H et al. used PTR in article [11] to evaluate the thermal and structural properties of silicone rubber insulators, providing a more in-depth understanding of the aging process. In article [12], PTR was used to quantitatively characterize the degree of aging of silicone rubber insulators in field operation, and it was proposed that the thermal diffusivity and the thickness of the aging layer could be used as its index. Article [13] proposed a field-dependent pollution model for determining the strength of the electric field on the surface of outdoor polymeric insulators, which can help to predict the formation of dry bands on the surface of outdoor polymeric insulators and the initiation of electrical discharges on the polymeric surface. Localized leakage currents may generate voltage peaks of up to 1000–5000 volts at insulator dead-ends. Article [14] focused on the online-condition monitoring of leakage currents in localized areas and the leakage-current effect to assess the severity of contamination on the surface of composite insulators. Compared to the continuous evolution of silicone rubber aging-detection methods, the progress of studying the influence of environmental factors on the aging process of silicone rubber has been slow.

The operating environment of silicone rubber insulators has a great influence on their aging process. In articles [15,16,17,18], the aging performance of silicone rubber composite insulators in coastal and inland tropical environments, arid climates, and the semihumid warm temperate regions of China were studied, respectively, and the embodied aging degree and aging rate were shown to differ. Among the environmental factors, the influence of acidity and alkalinity on silicone rubber composite insulators is particularly obvious. Article [19] studied the pollution performance of silicone rubber insulators by acid rain and concluded that acid rain accelerates the aging of silicone rubber; Verma AR et al. studied the aging of silicone rubber insulators accelerated by acid rain and their corrosion resistance in article [20], and the results showed that the surface of silicone rubber insulators corrode seriously in acidic environments, and the aging process is fast. Article [21] studied the effects of sulfuric and nitric acid solutions, as well as sulfate and nitrate solutions, in acid rain on silicone rubber; however, how to defend against the corrosion of composite insulator silicone rubber in acidic environments has not been studied in depth, and the indices and focus are not clear. Therefore, it is of practical importance to study the performance characteristics of silicone rubber used in acidic environments. Our team believes that different formulations of silicone rubber should be used in different acidic and alkaline environments, and is currently exploring the antiaging performance of different formulations of silicone rubber in acidic environments.

In this paper, we use the acid-resistant silicone rubber developed by our team to carry out a one-year natural aging test in a natural acidic environment. To verify its effectiveness, we also carried out a comparison test for the common-type silicone rubber. After carrying out dry flash and pollution flashover tests on it after one year of natural aging, and testing its hydrophobicity at the same time, we obtained the aging of the two types of silicone rubber in an acidic environment by analyzing the test results of scanning electron microscopy, Fourier transform infrared spectroscopy, X-ray energy-dispersive spectrometry, and by measuring the dielectric parameters of the two specimens; finally, we discuss the comparison between the common-type and the acid-resistant silicone rubber. Acid-resistant silicone rubber has a promising application in acidic areas. After the complete laboratory validation of the acid-resistant silicone rubber, the product can be introduced to the market, and it is believed that great economic benefits can be generated.

## 2. Materials and Methods

### 2.1. Experimental Samples

**Figure 1:**
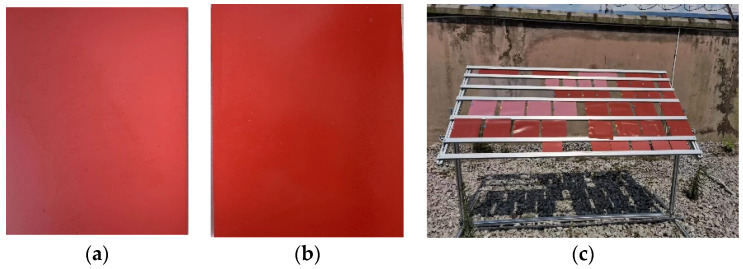
Silicone rubber sheets suspended in acidic environments. (**a**) General silicone rubber; (**b**) Acid-resistant silicone rubber; (**c**) Hanging site of test sample.

The samples in this paper are the same batch of new common and acid-resistant silicone rubber test products placed in a natural acidic environment and aging for one year (as shown in Figure 1). The specifications are 15 × 12 × 0.65 (note: length and width error ±1.0, height error ±0.10, in cm) of rectangular silicone film. The results of the analysis of the rainwater samples from this naturally acidic environment showed a pH of 4.10, with SO_4_^2−^, F^−^, Cl^−^, NO_3_^−^, and NO_2_^−^ as the main anions. The test samples are divided into four types according to the model and aging time: new common silicone rubber, aged-one-year common silicone rubber, new acid-resistant silicone rubber, aged-one-year acid-resistant silicone rubber.

### 2.2. Experimental Method

In order to comprehensively test the aging-state indexes of both types of silicone rubber sheets with a limited number of specimens, the tests in this paper need to use a variety of different and sequential methods to test them. For the naturally aged specimens, the natural dirt attached to the surface needs to be undamaged before the pollution flashover test, so the test sequence must be determined and strictly implemented before the test.

The test sequences were as follows: the industrial frequency (I.F) dry flashover test, hydrophobicity test (the spray method and static contact angle method), I.F. pollution flashover test, and microscopic test (including scanning electron microscopy, Fourier infrared spectroscopy, X-ray energy-spectrum analysis, and the measurement of dielectric parameters). In particular, in order to analyze the effect of settling the fouling on the performance of the silicone rubber, a water-repellency test was conducted before and after the pollution flashover test on the silicone rubber samples aged one year; in order to make the pollution flashover test data comparable, the new silicone rubber samples were manually fouled before the pollution flashover test, so that the surface fouling was the same as that of the silicone rubber samples aged one year.

**Figure 2:**
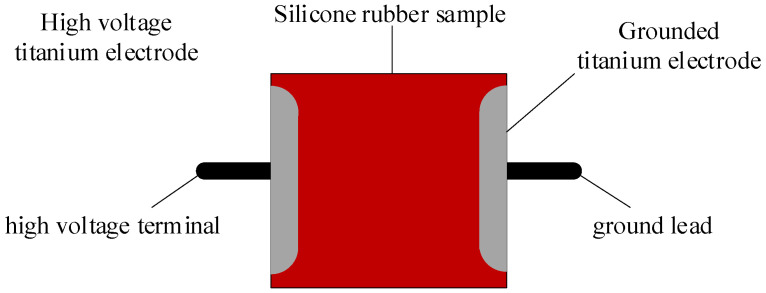
Electrode arrangement for flashover voltage test.

**Figure 3:**
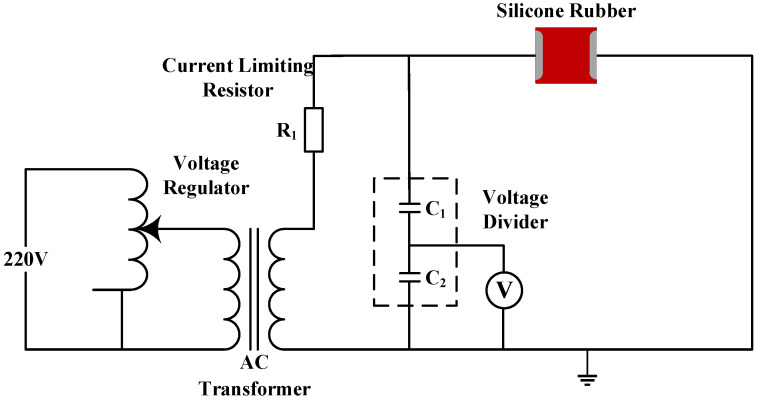
Test circuit.

- Electrical characteristics test: In this paper, the specimen is tested in the Chongqing University high-voltage laboratory for the I.F. dry flashover test and the I.F. pollution flashover test, and the test voltage is provided by the YDTW-100kVA/100kV AC transformer (from China Yangzhou Xinyuan Electricity Co., Yangzhou, China) to meet the test requirements. Figure 2 shows the electrode arrangement of the flashover voltage test, and Figure 3 shows the wiring schematic of the flashover test. During the test, the silicone rubber is placed horizontally, the high-voltage electrode is connected to the high-voltage end of the power supply through the protective resistor, the grounding electrode is directly grounded, and the flashover voltage is recorded through the capacitive voltage divider.
  Before the flash test is conducted, the test article needs to be pretreated. The new test product will be cleaned as thoroughly as possible with anhydrous ethanol, and then rinsed with a large amount of distilled water and dried for 24 h. Before the pollution flashover test, the new test product will be manually coated with dirt, so that the salt density and gray density of the surface are the same as the test product after one year of aging. The dry flashover and pollution flashover voltages of the silicone rubber specimens were measured by the uniform step-up method according to IEC 60060-1:2010 for insulators and the flashover voltage test method [22]. In order to ensure the accuracy and reliability of the flashover voltage data, and to eliminate the influence of chance, three specimens were taken for each sample, and three to five repetitions of the test were performed for each specimen. Take the average value of 9 voltages with the average error of not more than 10% and the relative deviation of not more than 5% as the average flash voltage of the test product. The average flashover voltage and the relative deviation are calculated as follows:
  (1) $$U_{f}=\frac{\sum_{i=1}^{m}U_{i}}{m},$$
  (2) $$\sigma \%=\sqrt{\frac{\sum_{i=1}^{m}{\left(U_{i}-U_{f}\right)}^{2}}{m}}\cdot \frac{100\%}{U_{f}}$$
  where *m* is the number of repeated tests (*m* = 9), *U_f_* is the average flashover voltage, and σ% is the relative deviation.

- Hydrophobicity test: The spray method is a method to initially classify the hydrophobicity of a material into 6 grades by spraying water on the surface and observing the state of the water droplets, with reference to IEC/TA 62073 and STRI Guide [23,24], and the hydrophobicity grade from 1 to 6 is gradually reduced. The static contact angle method: The optical-contact-angle measuring instrument commonly used to test the static contact angle is not applicable in this paper. This is due to the fact that the static contact angle measurement is required once before and after the pollution flashover test for the test article after one year of aging, and the natural dirt on the test article cannot be damaged before the test, which makes it impossible to cut the test article into the specifications needed for the measuring instrument to measure. In order to complete all experiments well within the constraints of the limited test items, the following methods are used in this paper: three samples of each silicone rubber were taken, and 10 drops of water with a volume of 10 μL were placed on each sample and photographed with a high-resolution camera; finally, the static contact angle θ of each water droplet was obtained by processing the photos with MATLAB, and the average value was recorded as the *θ_av_* of the sample.
- Scanning electron microscope (SEM): In this paper, the specimens were scanned by an environmental scanning electron microscope manufactured by Thermo Fisher, MA, USA, to investigate the microstructural differences. Due to the extremely poor electrical conductivity of the silicone rubber material, the specimens needed to be sprayed with 60 s gold before scanning.
- Fourier transform infrared spectroscopy (FTIR): A Nicolet iS50 Fourier transform infrared spectrometer from Thermo Fisher, MA, USA, was used to measure the infrared spectra of the specimens, and the test range of the equipment was from 400 to 4000 cm^−1^.
- X-ray energy-dispersive spectrometer: An ESCALAB 250Xi multifunctional X-ray photoelectron spectrometer from Thermo Fisher, MA, USA, was used to perform the X-ray spectroscopy on the flake specimens.
- Dielectric parameters: In this paper, we used the Alpha-Aconcept 80 broadband dielectric spectrum analyzer from Novecontrol Technologies, Montabaur (Germany), and the Agilent high-frequency analyzer to measure the dielectric parameters of specimens in the frequency range of 3 μHz to 40 MHz.

## 3. Results

This section may be divided by subheadings. It should provide a concise and precise description of the experimental results, their interpretation, as well as the experimental conclusions that can be drawn.

### 3.1. Surface Condition

**Figure 4:**
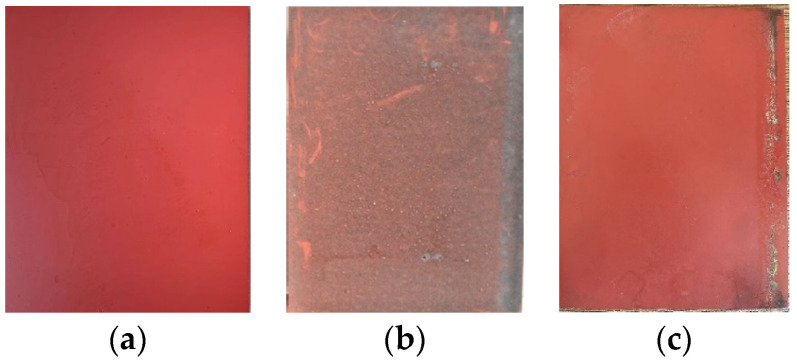
Common silicone rubber surface condition. (**a**) A new sample; (**b**) A sample after one year of aging; (**c**) A sample after one year of aging after cleaning.

**Figure 5:**
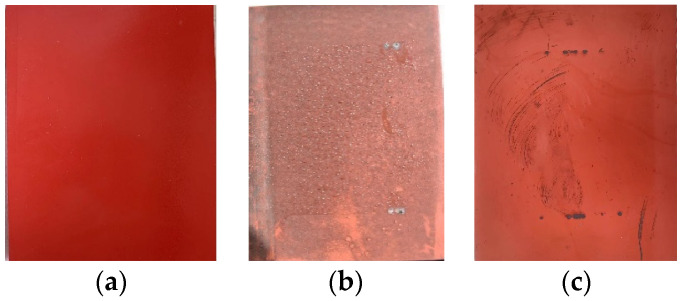
Acid-resistant type silicone rubber surface condition. (**a**) A new sample; (**b**) A sample after one year of aging; (**c**) A sample after one year of aging after cleaning.

The situation of the silicone rubber sheet after one year under acidic conditions is shown in Figure 4 and Figure 5. It can be seen from the figure that the new acid-resistant silicone rubber is darker than the common silicone rubber, and after one year of aging in the natural environment, a large amount of dirt has accumulated on the silicone rubber sheet, forming a dense layer of dirt on the surface, and the degree of dirt accumulation on the surface of the acid-resistant silicone rubber is lighter than that of the common type. After wiping out the dirt layer, the surface of the silicone rubber is obviously gray compared with the new test product, which is corroded by the acidic environment and has started to age.

### 3.2. Dry Flashover Voltage

This paper uses the I.F. dry flash test and the I.F. pollution flashover test on four samples, and the test results are shown in Table 1.

The results in Table 1 show that the dry flashover voltage of the common-type silicone rubber was lower than that of the acid-resistant type, with 7.2% and 7.6% lower for the new and aged-one-year samples, respectively. After one year of aging, the dry flash voltage of both types of silicone rubber decreased, while the dry flash voltage of the common silicone rubber decreased by 1.3% and the dry flash voltage of the acid-resistant silicone rubber decreased by 0.8%.

### 3.3. Hydrophobicity

In accordance with the method introduced in Section 2.2, the silicone rubber was subjected to the spray test and static contact angle test. In particular, for the silicone rubber aged one year, a static contact angle test was conducted again after cleaning with degreasing cotton and anhydrous ethanol, and the test results are shown in Table 2.

The results of the current study show that the static contact angle cut-off value for a good surface hydrophobicity of silicone rubber insulation material is 90°; that is, when the static contact angle of the specimen θ_AV_ > 90°, the surface hydrophobicity of the material is considered excellent, and vice versa, the material is considered hydrophilic. The test results in Table 2 show that the static contact angle of all the test products in this test is greater than 90°, and it can be considered that the hydrophobicity is excellent.

The data show that, after one year of natural aging, the surface static contact angles of the common and acid-resistant silicone rubber decreased by 4.3% and 3.7%, respectively, and the static contact angle on the surface of the new common silicone rubber and the samples aged for one year are 6.0% and 8.2% higher than that of the new acid-resistant silicone rubber and the samples aged for one year, respectively. In our experiments we also measured the static contact angle of the naturally aged silicone rubber before and after cleaning, and found that the static contact angle was greater when the silicone rubber was fouled.

This paper concludes that the presence of a coating on the surface of the acid-resistant silicone rubber is the reason why its static contact angle is greater than the static contact angle on the surface of the common-type silicone rubber under the same working conditions. The coating also protects the surface of the silicone rubber during the natural aging process, and the experimental results show that the static contact angle of the surface of the acid-resistant silicone rubber decreases to less than that of the common silicone rubber after one year of aging. Therefore, keeping the surface of the silicone rubber flat is an important means of maintaining the water repellency of the silicone rubber. The reason for the greater static contact angle of the silicone rubber with dirt is that, after the natural accumulation of dirt, the surface forms a uniform and dense layer of dirt.

### 3.4. Pollution Flashover Voltage

According to the I.F. pollution flashover test method mentioned in Section 2.2 for all four test products, the test results are shown in Table 3.

The results in Table 3 show that the pollution flashover voltage of the new acid-resistant silicone rubber is 7.6% higher than that of the common type, and the pollution flashover voltage of both types of silicone rubber decreases to different degrees after one year of aging, with the common type decreasing by 20.0% and the acid-resistant silicone rubber decreasing by only 14.8%, while the pollution flashover voltage of the acid-resistant silicone rubber is 13.4% higher than that of the common type after one year of aging. From the test data, it can be seen that the performance of the acid-resistant silicone rubber in terms of the pollution flashover voltage is better than that of the common silicone rubber before and after aging, while the aging speed is slower.

## 4. Discussion and Analysis

This chapter focuses on microscopic tests on silicone rubber samples and aims to corroborate the test results with each other with the test results in Section 3 of this paper.

### 4.1. SEM Analysis

In order to explain the changes in the water repellency and aging degree from the microstructure of the silicone rubber surface, this paper uses SEM to analyze the silicone rubber by magnifying 2000 times and 5000 times.

**Figure 6:**
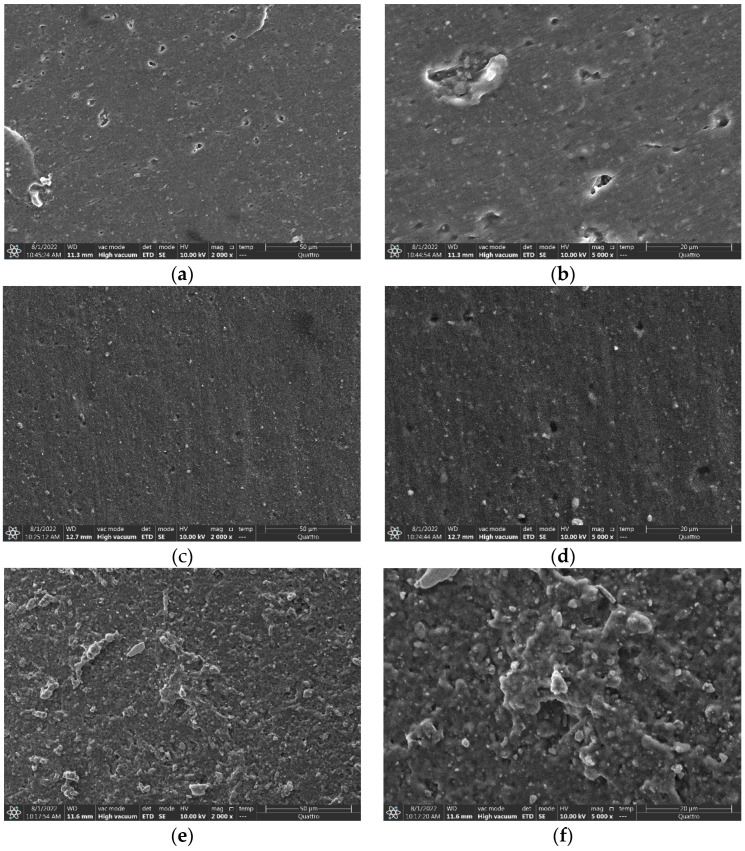

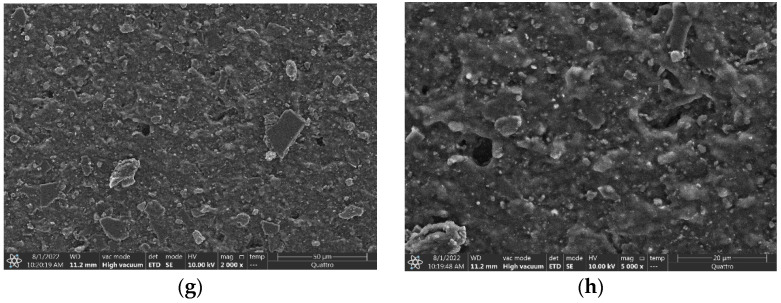
Surface micromorphology of silicone rubber sample. (**a**) New common type (×2000 times); (**b**) New common type (×5000 times); (**c**) New acid-resistant type (×2000 times); (**d**) New acid-resistant type (×5000 times); (**e**) Aged-one-year common type (×2000 times); (**f**) Aged-one-year common type (×5000 times); (**g**) Aged-one-year acid-resistant type (×2000 times); (**h**) Aged-one-year acid-resistant type (×5000 times).

The microstructure diagram of the surface of the specimen is shown in Figure 6. From the information in the figure, it can be seen that there are only tiny bubble holes on the surface of the two new silicone rubber products generated during the production of the trial products, and there are no large cracks and holes. Comparing (b) and (d), it is found that the edges of the bubble holes of the common-type silicone rubber new products are sharper than those of the acid-resistant type, but comparing (a) and (c), it can be found that the surface of the two types of silicone rubber are smooth, and the acid-resistant type of silicone rubber shows more undulations on the electron microscope photographs. Therefore, it is believed that the surface of the new normal type of silicone rubber is flatter than the acid-resistant type of silicone rubber. The surface of the two types of silicone rubber after one year of aging basically remained flat, and no obvious cracks had yet appeared; the coating on the acid-resistant silicone rubber would have the following effects on the microstructure of the silicone rubber surface: blunting the edges of the holes of the specimen, uneven painting leading to a protruding and relatively uneven silicone rubber surface, protecting the silicone rubber body, and the environment needed to destroy its coating before further oxidation of the silicone rubber surface could be carried out.

The results of the current study show that the microstructure of the surface of the silicone rubber material affects its water-repellent properties to a large extent, in which the roughness and porosity of the surface are the main factors affecting the static contact angle of the silicone rubber surface. Combined with the test results in Table 2, it can be concluded that, due to the presence of the coating, the roughness of the common-type silicone rubber is larger than that of acid-resistant-type silicone rubber, and the static contact angle is smaller. However, analyzing the aging time so far, the surface structure of the two types of silicone rubber has not been damaged more seriously, so its static contact angle can be kept in a higher range and its hydrophobicity is better.

### 4.2. FTIR Analysis

**Figure 7:**
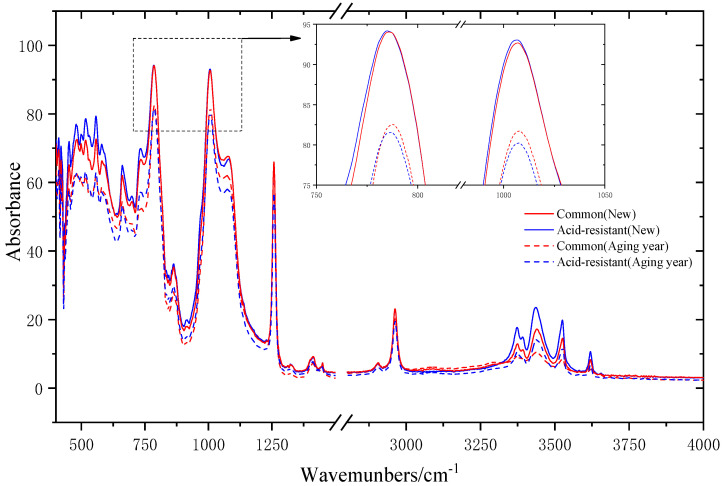
Infrared spectrum of the silicone rubber sample.

Different organic polymers produce different characteristic spectra in the infrared region, making infrared spectroscopy an important tool for studying polymer structures. The different characteristic groups corresponding to their resulting characteristic spectra are given in Table 4 [25,26]. In this paper, the infrared spectra of all four specimens were measured as a reference to comprehensively analyze the chemical structure changes occurring in different types of silicone rubber after natural aging. The test results are shown in Figure 7.

From Figure 7, it is clear that:

1. The main peaks of FTIR spectra of the silicone rubber sheets of different models and aging years remained basically the same; no new peaks were generated, the original peaks disappeared, and the corresponding bands of the peaks were not displaced, indicating that the chemical bonding composition of the silicone rubber surface remained basically the same, and no increase or decrease occurred;
2. The intensity of the Si−(CH_3_)_2_ absorption peaks located in the 840~790 cm^−1^ band from top to bottom are new silicone rubber of two types, common silicone rubber aged for one year and acid-resistant silicone rubber aged for one year, which are consistent with the performance results of the water-repellency test of the specimen. The Si−O−Si absorption peaks of the new silicone rubber sheet and the silicone rubber sheet aged for one year in the band of 1100~1000 cm^−1^ are basically the same, which indicates that the two types of silicone rubber sheets aged for one year have the same performance of keeping silicon small molecules from migrating and being lost. The C−H absorption peaks in the bands of 840−790 cm^−1^, 1270−1255 cm^−1^, and 2960 cm^−1^ also showed a significant simultaneous decrease. The decrease in the C−H bond content was mainly due to the fracture of the −CH_3_ functional groups in the silicone rubber;
3. The O−H bonds of the four test grades in the 3700~3200 cm^−1^ band showed obvious stratification, from top to bottom; they were the new acid-resistant silicone rubber, new common silicone rubber, aged-one-year acid-resistant silicone rubber, and aged-one-year common silicone rubber, indicating that the number of O−H bonds of the new acid-resistant silicone rubber was larger than that of the common type, which is consistent with the static contact angle. That is, the −OH content is high when the static contact angle is large. The intensity of the absorption peak of the O−H bonds decreased with the increase in the aging of the silicone rubber. Therefore, the large number of O−H bonds on the surface of the acid-resistant silicone rubber will help to delay the aging of the silicone rubber.

### 4.3. X-ray Energy-Dispersive Spectrometer Analysis

In order to further analyze the difference of the elemental content on the surface of the two types of silicone rubber and the change in the elemental content after natural aging, X-ray energy-spectrum analysis of silicone rubber was conducted in this paper to obtain the relative elemental content on the surface of all four test samples. According to the test results, comparisons were made between two types of new silicone rubber products, between two aging degrees of common-type silicone rubber, and between two aging degrees of acid-resistant-type silicone rubber, using the former surface of C, O, and Si, three elements’ relative contents as the benchmark, to visually show the differences and changes in the relative content of the elements on the surface of the silicone rubber, as shown in Table 5.

The test results show that the relative contents of the three index elements of the two types of new silicone rubber are basically the same; after one year of natural aging, the C and Si element contents on the surface of both types of silicone rubber have significantly decreased, and the relative content of the O element has increased. From the data in the table, it can be seen that the C element retention ability of the acid-resistant silicone rubber is stronger than that of the common type, the oxidation resistance is slightly stronger than that of the common type, and the Si element retention ability is the same as that of the common type, which is consistent with the FITR test results.

The data in Table 5 show that:

1. The surface oxygen element content of the new acid-resistant silicone rubber is higher than that of the common type. This is due to the presence of a large number of O−H bonds in the coating present on the surface of the acid-resistant silicone rubber, as evidenced by FTIR tests, while the content of both carbon and silicon elements is basically the same;
2. After one year of natural aging, the small silicone molecules in the silicone rubber (carbon and silicon elements are mainly silicone molecules) begin to migrate to the surface of the fouling layer, resulting in the carbon and silicon content of the silicone rubber beginning to fall, while the content of inorganic flame retardants (Al(OH)_3_) in the material remains unchanged, leading to a rise in the relative content of oxygen elements;
3. Acid-resistant silicone rubber has a better ability to resist oxidation and to keep small silicone molecules from migrating than the common silicone rubber.

### 4.4. Dielectric Parameters Analysis

Dielectric properties are the most basic physical properties of dielectrics, and the study of dielectric parameters of the common and acid-resistant silicone rubber is of great significance to assess their sexual differences and aging degree. In this paper, the dielectric parameters of all four samples were measured and their dielectric loss angles at 50 Hz were derived, as shown in Table 6.

From the table, it can be seen that the acid-resistant silicone rubber increases by 19.3% in the dissipation angle after natural aging, while the common-type silicone rubber increases by 62.1%. The dielectric loss of the new common-type silicone rubber is 8.9% larger than that of the acid-resistant type, and this figure becomes 33.0% after the silicone rubber undergoes natural aging. This is due to the chemical degradation of silicone rubber after natural aging, which leads to physical defects on its surface. According to the theory of dielectric polarization, it can be assumed that the dielectric loss angle of the silicone rubber samples increases with aging, so it can be assumed that the aging rate of the acid-resistant silicone rubber in dielectric properties is less than that of the normal-type silicone rubber.

## 5. Conclusions

This paper takes samples of acid-resistant silicone rubber and common silicone rubber with different aging years as the object of study, starting with the study from the aging degree of the two types of silicone rubber, and verifies them through microscopic tests, and comes to the following conclusions:

(1) The dry flashover voltage and pollution flashover voltage of the acid-resistant silicone rubber are greatly improved compared to those of the common-type silicone rubber, while the acid-resistant silicone rubber’s resistance to dirt and aging is also stronger. In terms of hydrophobicity, the acid-resistant silicone rubber also shows better performance, but due to the short aging time, both types of silicone rubber specimens show good hydrophobicity;
(2) From the results of the SEM, in the preaging period, the chemical degradation of common-type silicone rubber had not yet penetrated into the interior of the silicone rubber, and only produced tiny debris at the surface; whereas the acid-resistant silicone rubber had the surface of the coating oxidize first, which protected the silicone rubber body, and this phenomenon also created an acid-resistant silicone rubber with a stronger hydrophobicity;
(3) The results of the three methods of the FTIR, X-ray energy-spectrum analysis, and dielectric parameter testing on the silicone rubber were all consistent with the results of the electrical property testing and SEM, verifying the ability of the acid-resistant silicone rubber to retard aging in terms of microscopic characteristics;
(4) Considering the growth of aging time, common-type silicone rubber is not protected by an acid-resistant coating, resulting in a faster surface-oxidation speed in an acidic environment, leading to acidic large cracks and resulting in holes, rough protrusions, and a substantial degradation of performance;
(5) The study in this paper has some objective limitations. The aging time of the samples in the natural environment was only one year, and future studies need to observe their performance in longer time scales, as well as the performance characteristics of alkali-resistant silicone rubber in alkaline environments.

## Figures and Tables

**Table 1:** Experimental results of the dry flashover voltage of the silicone rubber.

Type	Aging Years (Year)	Average Dry Flashover Voltage (kV)	Relative Deviation
Common	0	46.7	1.63%
	1	46.1	1.59%
Acid-resistant	0	50.3	1.37%
	1	49.9	0.85%

**Table 2:** Test results of the surface hydrophobicity of the silicone rubber.

Type	Aging Years (Year)	with Dirt		after Cleaning	
		θ_AV_ (°)	HC Grading	θ_AV_ (°)	HC Grading
Common	0	/	/	115.2	1
	1	117.8	1	110.2	2
Acid-resistant	0	/	/	131.1	1
	1	134.9	1	126.2	1

**Table 3:** Pollution flashover test results of the silicone rubber.

Type	Aging Years (Year)	Average Pollution Flashover Voltage (kV)	Relative Deviation
Common	0	20.2	2.52%
	1	16.2	4.90%
Acid-resistant	0	22.9	3.30%
	1	18.6	2.99%

**Table 4:** Characteristic peaks of silicon rubber in IR analysis.

Functional Groups	Wavenumbers (cm^−1^)
Si(CH_3_)_3_	800~700
O−Si(CH_3_)_2_−O(Si−O)	840~790
Si−O−Si(Si−O)	1100~1000
Si−CH_3_(C−H)	1270~1255
CH_3_(C−H)	2960
O−H	3700~3200

**Table 5:** Comparison of the relative contents of surface elements between samples.

Type	Aging Years (Year)	Relative Content of C (%)	Relative Content of O (%)	Relative Content of Si (%)
Common	0	35%	26%	29%
	1	34%	32%	27%
Acid-resistant	0	36%	28%	29%
	1	35%	33%	28%

**Table 6:** Silicone rubber dielectric parameter test results.

Type	Aging Years (Year)	Dielectric Loss Angle	Rate of Change
Common	0	0.0393	/
	1	0.0637	+62.1%
Acid-resistant	0	0.0358	/
	1	0.0427	+19.3%

## Data Availability

Data presented in this study are available on request from the first author.
